# State of affairs and future challenges in laboratory medicine in Spain: an analysis of the Spanish Society of Laboratory Medicine (SEQC^ML^)

**DOI:** 10.1515/almed-2023-0013

**Published:** 2023-03-06

**Authors:** Imma Caballé, Antonio Buño, Francisco A. Bernabeu, Francesca Canalias, Antonio Moreno, Mercè Ibarz, José Puzo, Concepción González, Álvaro González

**Affiliations:** Laboratorio Catlab, Terrassa, Barcelona, Spain; Department of Clinical Laboratory Analytics, Hospital Universitario La Paz, Madrid, Spain; Servicio de Bioquímica – Clinical Laboratory, Hospital Universitario Puerta de Hierro Majadahonda, Madrid, Spain; Laboratori de Referència d’Enzimologia Clínica, Departament de Bioquímica i Biologia Molecular, Universitat Autònoma de Barcelona, Cerdanyola del Vallès, Spain; Service of Clinical Laboratory Analysis, Hospital de Meixoeiro, Vigo, Spain; Clinical Laboratory ICS Lleida, Hospital Universitario Arnau de Vilanova de Lleida, Lleida, Spain; Service of Clinical Laboratory Analysis and Biochemistry, Hospital Universitario San Jorge, Huesca, Spain; Service of Clinical Biochemistry, Hospital Universitario Virgen Macarena, Seville, Spain; Service of Clinical Biochemistry, Clínica Universidad de Navarra, Madrid, Spain

**Keywords:** future challenges, laboratory medicine, sector status

## Abstract

**Objectives:**

Laboratory Medicine is a crucial discipline that contributes to the diagnosis, management and monitoring of patients. This branch of medicine faces two major challenges: New technologies and increased demand. There is limited information available of the state of affairs in Laboratory Medicine in Spain. This study provides a picture of clinical laboratories and clinical laboratory professionals.

**Methods:**

The Spanish Society of Laboratory Medicine distributed a questionnaire among the 250 most representative centers (the ones with the largest volume of determinations and training programs), of which 174 (69.6%) returned the questionnaire providing data for 2019.

**Results:**

Laboratories were classified according to the number of determinations. In total, 37% identified themselves as small (<1 million determinations per year); 40% considered themselves medium-sized (1–5 million determinations per year) and 23% considered they were large laboratories (>5 million determinations). The level of specialization of laboratory physicians and laboratory performance were higher in large laboratories. Most requests (87%) and determinations (93%) corresponded to biochemistry and hematology. As many as 63% of physicians had an indefinite contract, and 23% were older than 60 years.

**Conclusions:**

Laboratory medicine is a consolidated discipline that is gaining relevance in Spain. It adds value to the diagnosis, prognosis and follow-up of diseases, and to treatment response monitoring. The results of this study will help us address challenges such as the need for specialized training for laboratory professionals; the emergence of technological innovations; exploitation of Big Data; optimization of quality management systems and patient safety.

## Introduction

Laboratory Medicine is a branch of medicine concerned with the quantitative determination or qualitative evaluation of any substance present in a biological fluid for clinical or research purposes. This determination or evaluation is known as diagnosis *in vitro*, since it is performed out of the body [1]. The results of laboratory tests are translated into information that is useful to improve the health status or wellbeing of individuals or of the population [2, 3]. The results of clinical laboratories are also used for the screening, prevention and early detection of diseases, as well as for the diagnosis, monitoring and prediction of response to treatment. It is estimated that 66% of clinical decisions are based on laboratory results [4, 5].

Despite the significant value and relevance that laboratory tests have for the diagnosis and management of patients, they only account for 3% of health expenditure in Spain [6]. Clinical laboratories face a growing demand for determinations, as a result of the increase in the incidence of chronic and infectious diseases in an ageing population [7]. In Europe, the value of the *in vitro* diagnostics market was estimated to reach 13,825 million in 2019, and is projected to exceed 18,000 million by 2027, at a compound annual growth rate of 4.5% [7].

Evidence about the current status of clinical laboratories in Spain is limited, as evinced by the lack of a national database. This study is intended to provide an overview of the state of affairs in clinical laboratories in Spain. This will be the first hallmark in the way to define strategies and actions aimed at improving clinical laboratory performance in the future.

The purpose of this study was to gather information on the activity of clinical laboratories in Spain, describe their portfolio of services and areas with the highest demand, and provide a picture of the situation of clinical laboratory professionals. Another objective was to provide information on research and training activities promoted by clinical laboratories. This initiative, launched by the Spanish Society of Laboratory Medicine, led to the release of the *White Book of Laboratory Medicine*. This paper provides a first overview and reports the most relevant findings [8].

## Materials and methods

### Research site selection

To obtain a general picture of clinical laboratories, a selection was performed of the 250 most representative laboratories in the list provided by the Spanish Ministry of Health, Consumption and Social Wellbeing in January 2020 [9]. Centers were considered representative if they were included in the National Portfolio of Hospitals, with priority having been given to large hospitals (those with the highest number of beds) with education and training activity (Supplementary Figure S1). A questionnaire was distributed among the selected centers.

### Variables of interest

An analysis was carried out of data for year 2019, where 55.9 million requests and 800 million determinations were performed. Data for subsequent years was not collected to prevent bias due to the COVID-19 pandemic.

Data collection included general laboratory data (services, ownership); information on the organizational and activity model (number of requests, continuing care model; equipment or consumable procurement); human resource data (types of contracts, employee demographics); information on education and training activity (number of faculty members and residents); quality management systems adopted; type of research led by the laboratory (dissertations, master’s degree final project) and level of process automation. Lastly, participants were asked to identify future challenges and priorities.

The data obtained were compared against data from the Spanish Federation of Healthcare Technology Companies (FENIN) 2018 report [10], the Ministry of Health [11, 12], and global data [13].

This study complied with Organic Law 3/2018 on the Protection of Personal Data Protection and Guarantee of Digital Rights. Data was treated as confidential and anonymous.

### Statistical analysis

Qualitative variables are expressed as total number and percentage. Quantitative variables are presented as mean or median and interquartile range. Statistical analysis was carried out using Excel with customized add-ins.

## Results and discussion

### Structure and model of clinical laboratories in Spain

**Figure 1:**
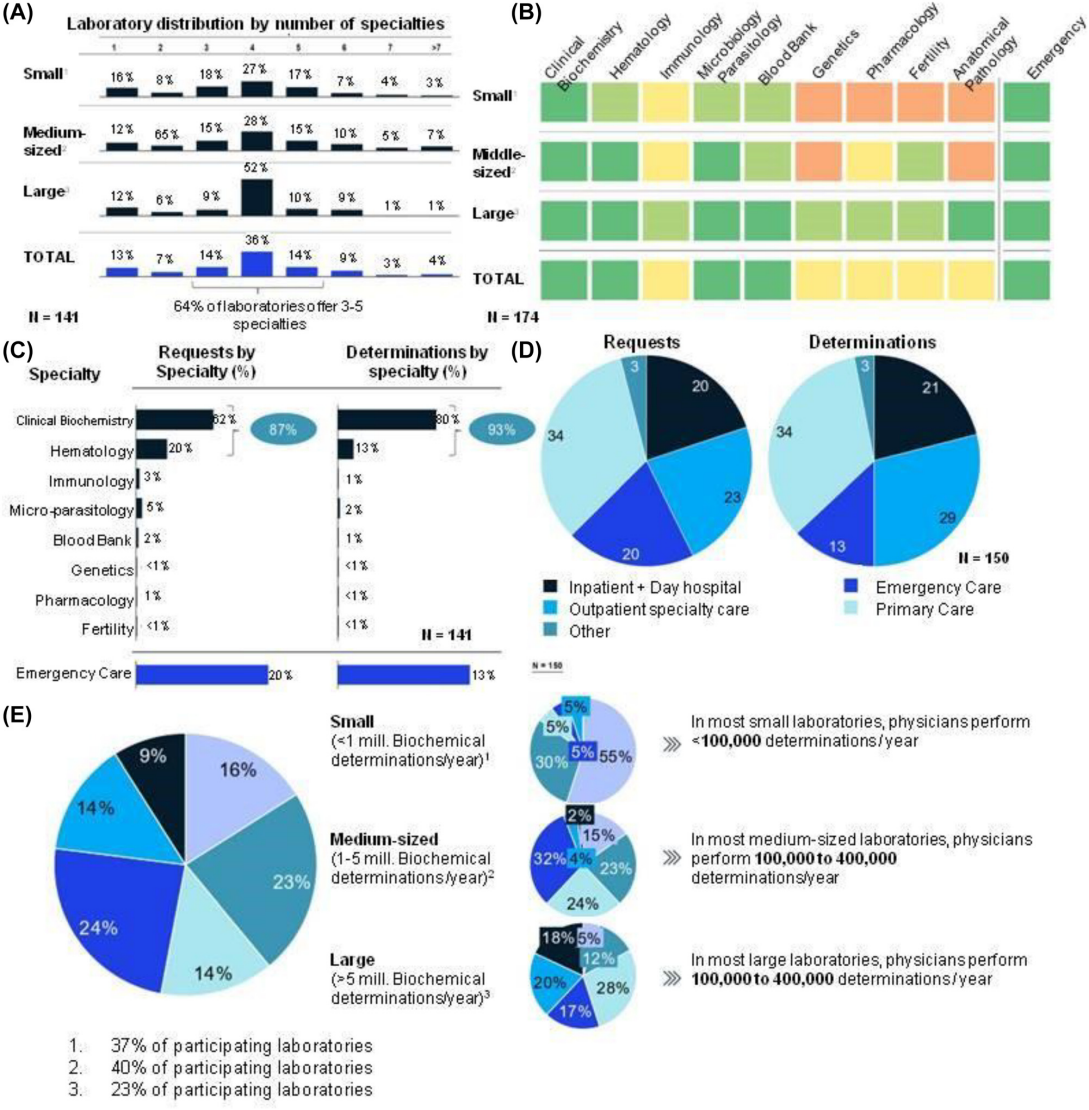
Sector model and activity. (A) Percentage of laboratories with the presence of a specific number of specialties; (B) specialties available in clinical laboratories by size and availability of an emergency service; (C) percentage of requests and determination by specialty; (D) requests and determinations by referring unit; (E) distribution of clinical laboratories by number of determinations by physician.

As many as 66% of the laboratories interviewed were general laboratories, whereas 90% only had one associated hospital. In total, 37% considered themselves a small laboratory (<1 million determinations per year); whereas 40% considered themselves medium-sized laboratories (1–5 million determinations per year); and 23% deemed they were large laboratories (>5 million determinations). Most laboratories had 3 to 5 specialties, with the smallest laboratories having the highest number of specialties (Figure 1A). This could indicate that organizational models were determined by the size of the laboratory, and that large laboratories are most oriented to large-scale diagnostic testing. Biochemistry, hematology, microbiology and a blood bank were available in the majority of centers (>60%). Immunology, pharmacology, andrology, reproduction and anatomical pathology services were available in half of the centers (Figure 1B). As many as 80% of centers had an integrated emergency service in the laboratory.

### Clinical laboratory activity in Spain

In relation to laboratory activity by specialty, clinical biochemistry and hematology accounted for most of requests (87%) and determinations (93%). In total, 20% of requests and 13% of determinations were urgent (Figure 1C). Most of requests and determinations were sent from Primary Care (34% for both) and Specialty Care (23 and 29% for requests and determinations, respectively). Requests from Specialty Care were the ones that included the highest number of determinations, with a mean of 16 determinations per request (Figure 1D). Requests from Primary Care included a mean of 15.5 determinations. Personnel performance based on the number of determinations by clinician was higher in large laboratories. Ninety-five percent of large laboratories performed 100,000 to 400,000 determinations per year per clinician (Figure 1E). Large laboratories, which perform large-scale testing, probably benefit from the higher level of specialization of their physicians, thereby resulting in higher performance. Globally, clinical laboratories could perform internally 97% of the determinations requested. This indicates that, to face the increasing demand, it is necessary that the capacity of clinical laboratories is improved.

### Management, strategy, procurement and hiring practices

**Figure 2:**
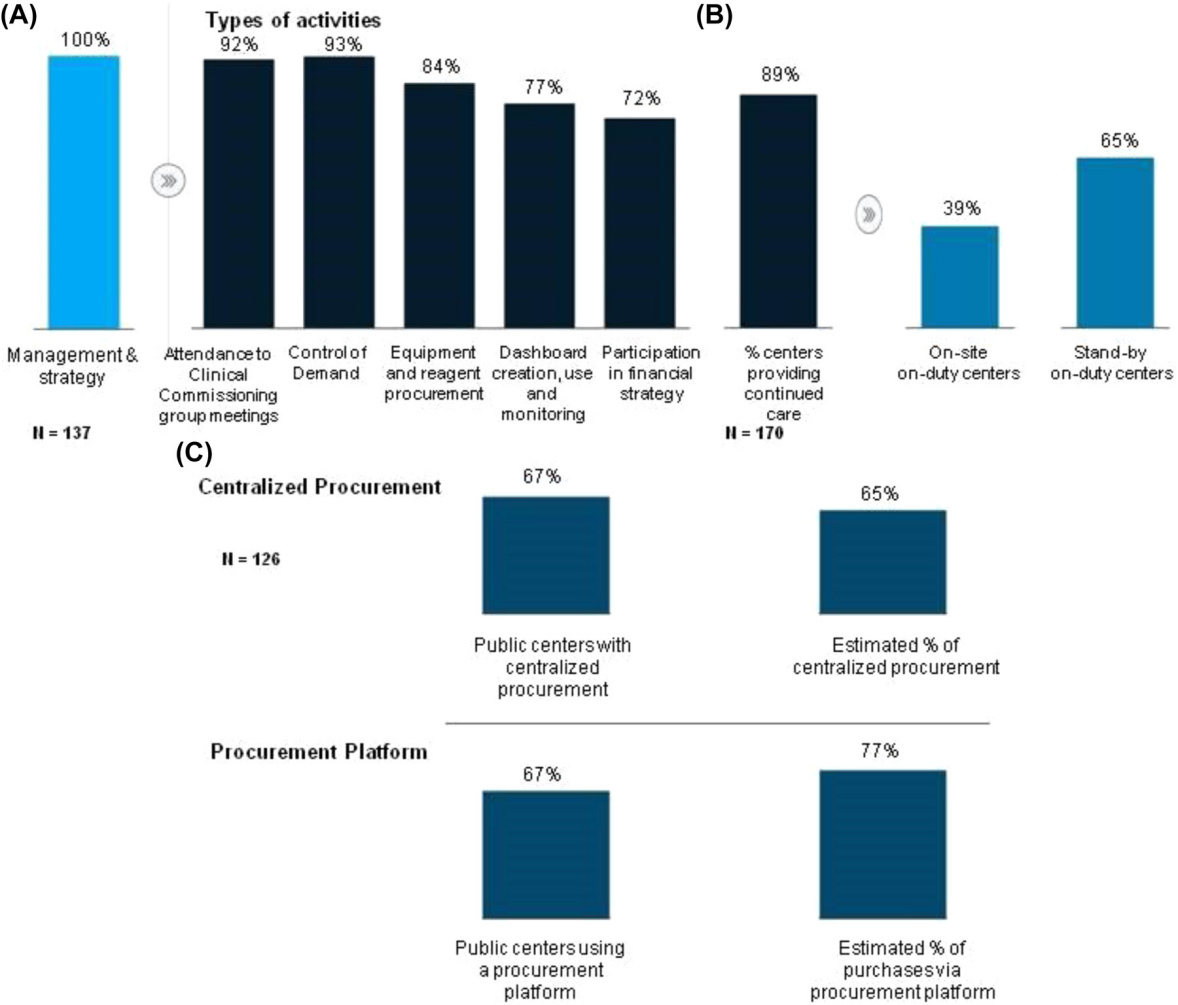
Management, strategy, procurement and hiring practices. (A) Percentage of laboratories that perform management and strategy activities and breakdown by main management and strategy activities; (B) continuing care model; (C) level of centralization of procurement.

All laboratories were involved in management or strategy activities. The most common activities included attendance to meetings of clinical commissioning groups (92%); control of demand (93%); and equipment and reagent procurement activities (84%) (Figure 2A). Eighty-nine percent of laboratories had a continuing care model, including stand-by duty shifts (65%) or on-site duty shifts (39%) (Figure 2B).

Procurement was centralized in 126 public centers via specific platforms. It is estimated that 65% of purchases were centralized (Figure 2C).

### Human resources

**Figure 3:**
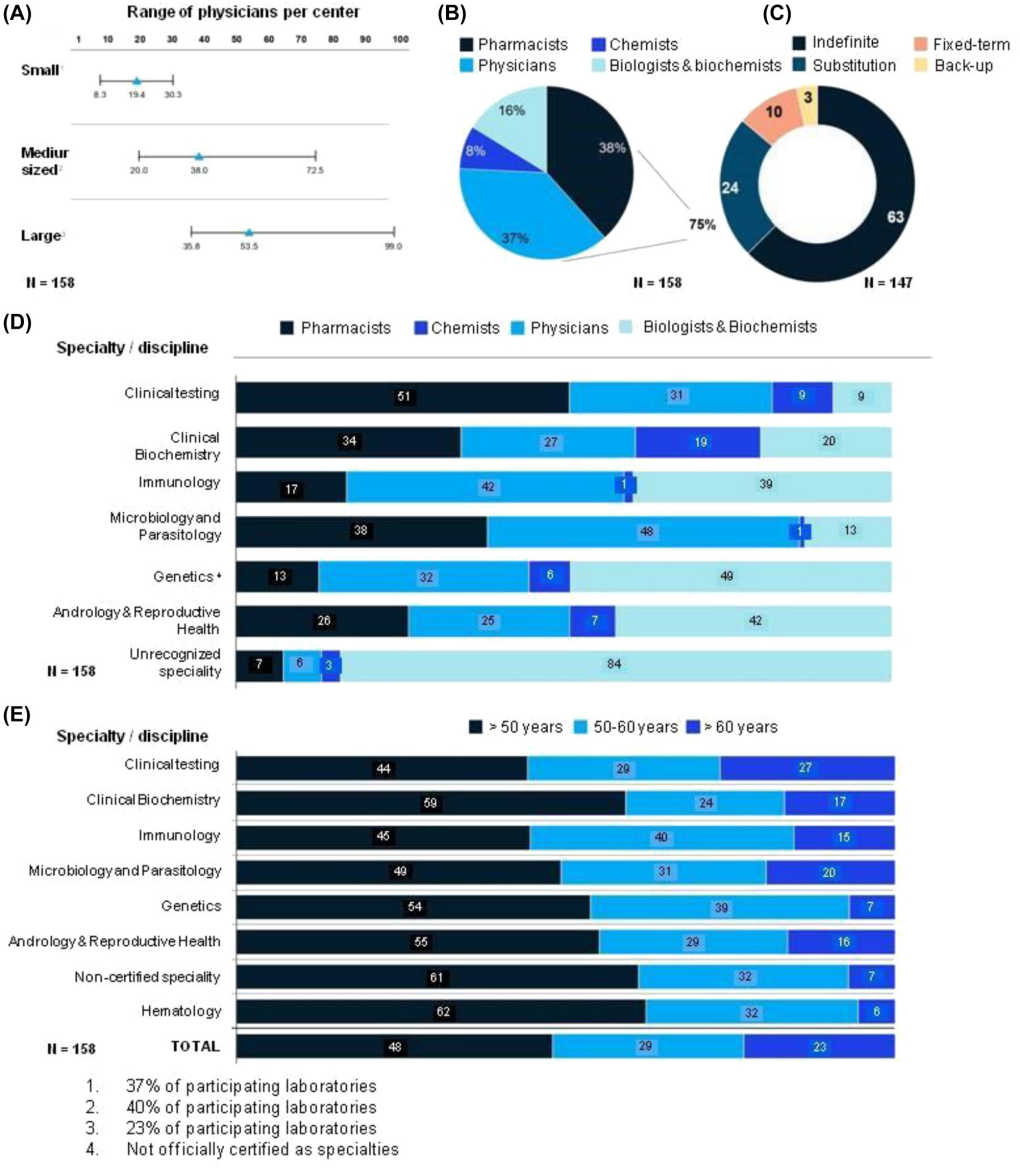
Human resources. (A) Number of physicians by laboratory and size, according to self-reported data; (B) distribution of the types of contracts in the participating laboratories; (C) distribution of certified specialists; (D) distribution of certified specialists by area of knowledge; (E) age distribution of graduate professionals by specialty.

There was a direct relationship between the number of physicians per center and the size of the center (Figure 3A). As many as 63% of physicians had an indefinite contract. Twenty-four percent had substitution contracts; 10% had fixed-term contracts; and 3% were back-up staff (Figure 3B). Hiring practices were influenced by geographic location. Catalonia, Navarra, Asturias and Galicia were the autonomous communities with the highest percentage (>70%) of physicians with an indefinite contract. In general, a relatively high percentage of professionals were full-time.

With regard to education and training, 38% of physicians had a Master’s degree in Pharmacy; 37% in Medicine; 16% in Biology and Biochemistry and 8% in Chemistry (Figure 3C). There were more physicians and pharmacists in classical specialties such as laboratory analysis (82%) or biochemistry (61%). In contrast, biologists and biochemists were more frequently found in areas such as genetics (49%), andrology and reproduction (42%) or other (84%) (Figure 3D).

In relation to the age of physicians in Spain, 23% were older than 60 years, which means that 20–25% of positions will need to be filled by 2024 (Figure 3E).

### Continuing training, education and research

**Figure 4:**
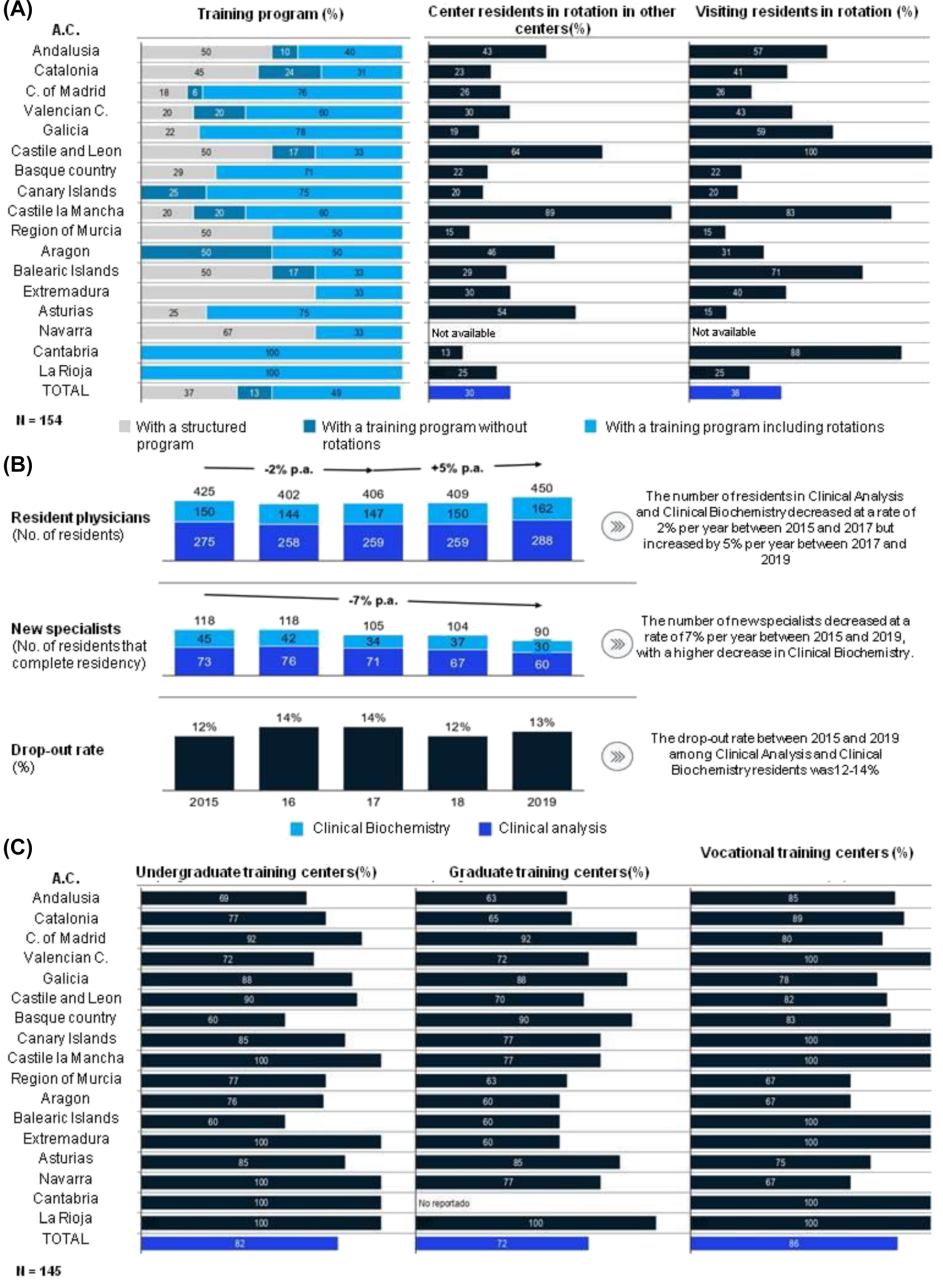
Lifelong learning, teaching and research. (A) Percentage of centers with structured training and education programs by autonomous community; percentage of rotation residents by autonomous community and percentage of rotation residents in the laboratory by autonomous community; (B) intern specialists, new specialists and drop-out rate in clinical analysis and clinical biochemistry; (C) Percentage of clinical laboratories that offer undergraduate, graduate and vocational training by autonomous community.

Most clinical laboratories (63%) had a structured education and training plan involving rotations (50%) or not (13%) (Figure 4A).

The number of internal medicine residents (known as MIR in Spain) decreased by 2% between 2015 and 2017, although it increased by 5% between 2017 and 2019, taking data from the National Health System as a reference (SNS) [12]. In the 2015–2019 period, the number of new specialists decreased at an annual rate of 7%. The specialties related to clinical laboratory medicine are among the specialties with the lowest demand. The drop-out rate was 12–14% (Figure 4B). These percentages indicate that these specialties are not attractive to new physicians. This may be explained by the low attention paid to laboratory medicine in medicine schools, and to limited interaction with patients in the clinical laboratory. This trend was not observed in other disciplines, such as biology or chemistry.

At national level, 82% of participants were undergraduate professors, 72% were graduate professors, and 86% were vocational education and training teachers (known as FP in Spain). The totality of the centers in the Valencian Community, Castile and Leon, the Canary Islands, Castile-La Mancha, the Balearic Islands, Extremadura and Cantabria were vocational training centers (Figure 4C). Likewise, 13.5% of clinical laboratory specialists worked as professors with different contractual modalities. Each professor in active service had published a mean of 13.5 papers in indexed journals in the preceding five years. Professors in large centers led a higher number of research projects. Forty-five percent of centers had a partnership with a research center or institute. In total, 18% of centers had research agreements with the industry, with a mean of three agreements per center.

The relevance that centers ascribe to knowledge, education and training is demonstrated by their intense research and education activity (undergraduate, graduate or vocational). Another proof is the sustained presence of internal residents in the centers and the availability of training programs for internal residents. In half the centers, structured training programs included rotations for internal residents. The intense research activity reported by the centers is a clear proof of the relevant role that research has in the participating centers. Innovation is central for health systems to be able to meet current and future challenges through the use of effective, state-of-the-art services.

### Regulatory framework and standards

**Figure 5:**
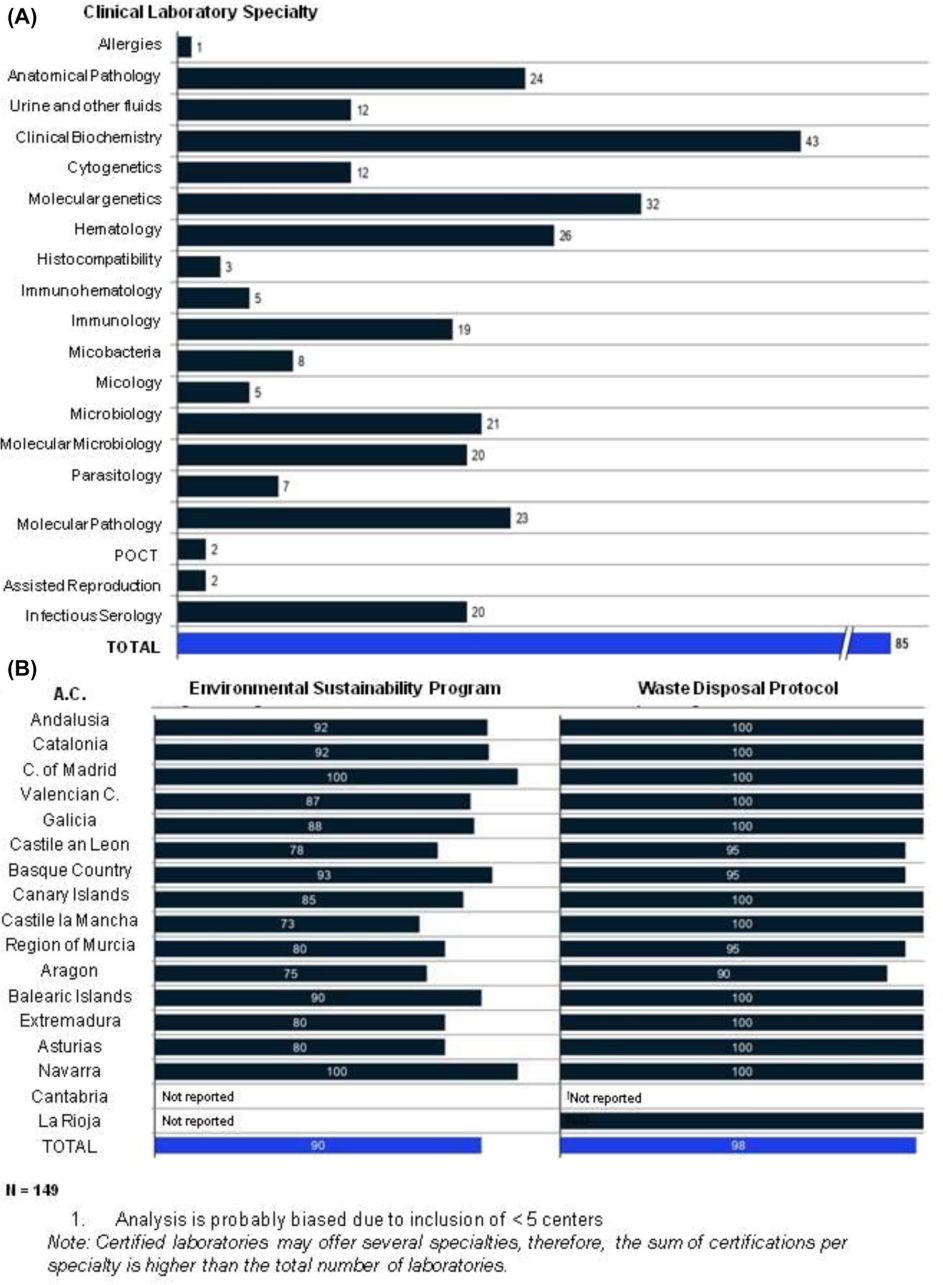
Regulatory framework and quality standards. (A) Number of UNE-EN-ISO 15189-certified centers by specialty reported by ENAC (May 2021); (B) percentage of centers with an environmental sustainability program and waste disposal protocol.

Only 49% of participants held ISO 9001 certification. According to the Spanish National Certification Agency (ENAC), in relation to Quality Management in Clinical Laboratories, only 85 laboratories had been granted UNE-EN ISO 15189 certification (Figure 5A) [14]. These results demonstrate that there is a lot of room for improvement in most of the centers in the field of procedure standardization and quality management.

With regard to environmental issues, 90% of laboratories had an environmental management program, and 98% had a waste disposal protocol (Figure 5B). These results evince laboratories’ commitment to environmental sustainability.

### Spanish laboratory medicine in the European context

In Spain, the average expenditure in *in vitro* diagnostic supplies was 1,033 million euros in 2019, vs. 2,161 million euros in Germany, 1,623 in France, and 859 in the United Kingdom [15]. Expenditure increased significantly In Spain between 2019 and 2020. These figures place Spain in a similar range as other European countries, with a mean expenditure of 22 euros per inhabitant. In contrast, the number of specialists per million inhabitants is considerably higher in Spain (53.3 specialists per million inhabitants), as compared to other European countries such as Germany (25.5 specialists per million inhabitants). It is worth mentioning that laboratory specialty is not available for pharmacists in the United Kingdom and Germany. Differences in laboratory technician training may also affect comparative analysis of the number of specialists.

### Trends and future challenges

The Spanish population is one of the most rapidly ageing in Europe. This phenomenon will translate into increased general morbidity rates and higher prevalence of chronic diseases. As a result, the demand for laboratory analyses will grow. Advances in molecular biology and genomics, along with the emergence of new scientific disciplines such as computation, data analysis and artificial intelligence, have brought about a bio-revolution that will change the way we understand laboratory medicine.

**Figure 6:**
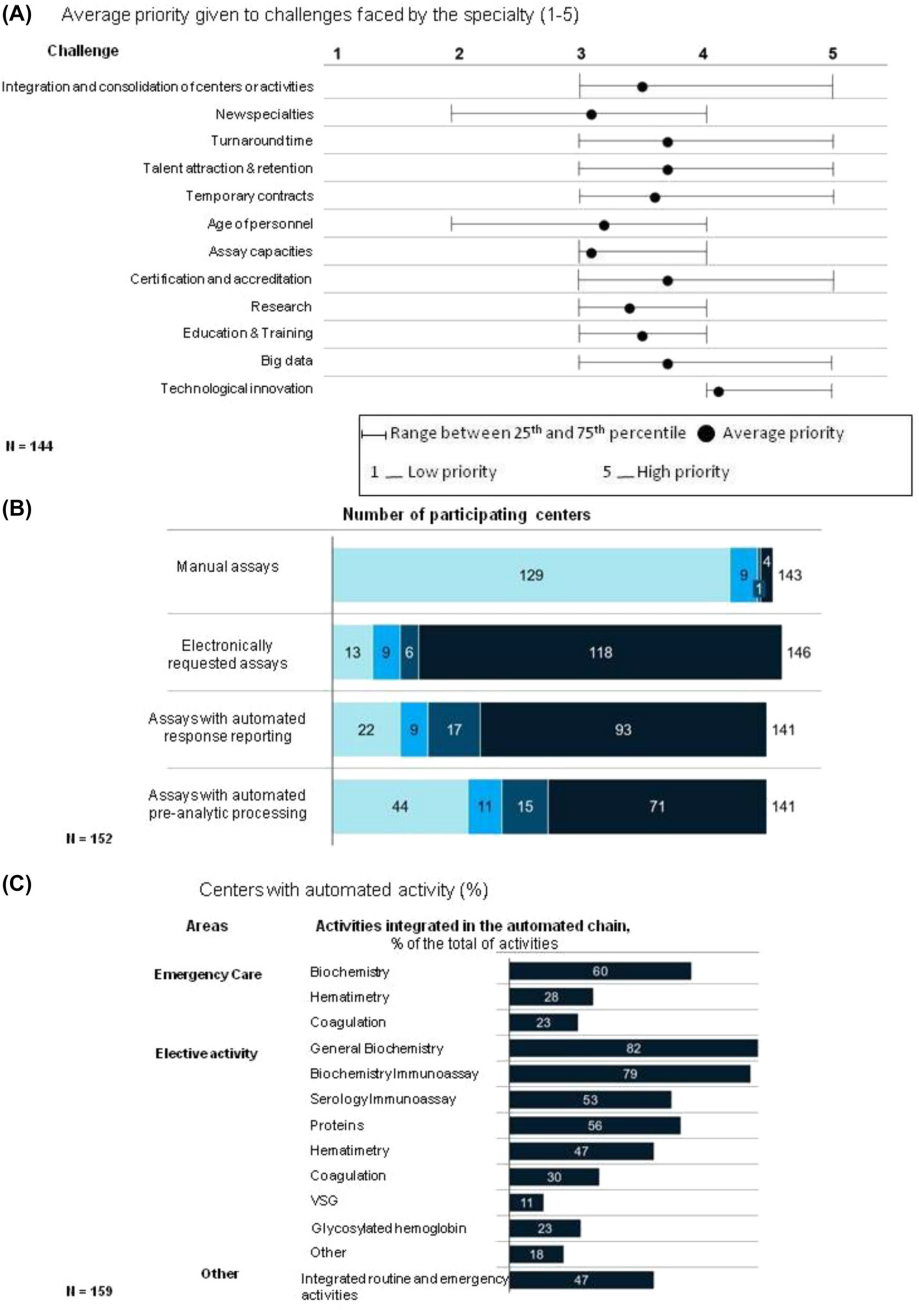
Future challenges. (A) Main challenges faced by the specialty, based on questionnaire responses; (B) level of process automation by type of test, based on questionnaire responses; (C) percentage of centers with an automated chain, based on questionnaire responses.

According to the participants, laboratory medicine faces various challenges, including technological innovation, Big Data analysis, provision of rapid response to patients, quality certification systems and talent attraction and retention (Figure 6A). Technological advances do not only improve the efficiency of processes, but also open new ways to meet user’s needs, manage and interpret big data and help laboratories provide a more efficient, safe and personalized service. As a transversal element, technology may play a pivotal role. The application of Big Data and Artificial Intelligence in the clinical laboratory will be crucial to clinical decision-making and health monitoring. Indeed, some initiatives have already been launched [16]. Participants have made great efforts to incorporate digital and automation technologies in their standard operating procedures. As many as 80% of participants received data electronically. Ninety percent of tests were automated, and two thirds (68%) had automated result reporting systems (Figure 6B, C).

## Conclusions

This study provides a complete picture of the state of affairs in laboratory medicine in Spain and paves the way for future actions aimed at helping the sector improve and face future challenges. However, this study provides a cross-sectional view, a snapshot in time. Longitudinal studies are needed to infer trends and anticipate sector needs more accurately. The results of this study show that the specialties of Clinical Laboratory and Clinical Biochemistry have merged into a single specialty, which forces us to reconsider the situation to make grades equivalent to those in other European countries.

Clinical laboratory staff does not only consist of physicians, as suggested in national estimations, but also includes pharmacists, chemists and biologists. Biologists are widely present in emerging sectors such as genetics. This added to the need to replace retiring professionals will make it necessary to increase the number of vacancies offered. Specialized training is pivotal to cope with the shortage of vacancies.

The impact of COVID-19 was not analyzed in this study. However, future studies should examine how the pandemic influenced the number of determinations and model implemented, with direct-to-consumer (DTC) diagnostic tests having an increasing weight in the volume of tests requested. Point-of-care testing may also increase significantly in the near future, as a result of advances in mobile health applications (m-Health). There are a variety of innovations in this field.

This study also reveals the need to provide an updated database of clinical laboratories to include them in national health statistics. A regulatory progress is necessary to align with the European context and advance in laboratory quality management certification practices.

Cooperation between national and international societies may facilitate access to education and training and promote the incorporation of young professionals to decision-making boards and committees. Cooperation may also improve access to grants and promote collaboration with medical research societies, in order to attract and retain new talent and boost innovation in our sector.

Laboratory medicine should expand its presence in the media to create awareness on its contribution to society. Moreover, laboratory medicine representatives should also strengthen ties with regional and national entities to enlist their support in making advancements and acquiring a more relevant position within the health system.

## Supplementary Material

Supplementary Material
