# Supplementary figures and images for: State of affairs and future challenges in laboratory medicine in Spain: an analysis of the Spanish Society of Laboratory Medicine (SEQCML)
Source: Adv Lab Med. 2023 Mar 6;4(1):70–80. doi: 10.1515/almed-2023-0013 (PMC10197191; doi:10.1515/almed-2023-0013)

**Suplementary Figure 1**. Participating centers by autonomous community and type of center.


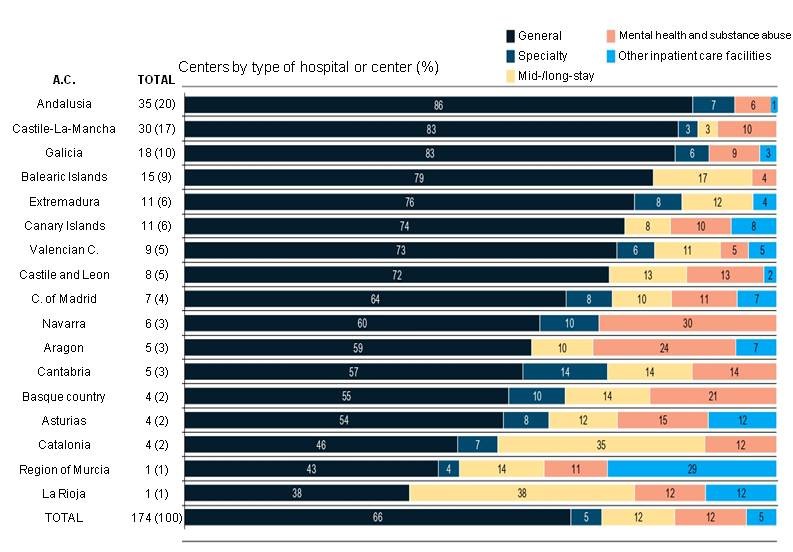

Supplement: Supplementary file 1 — Supplementary Material [file j_almed-2023-0013_suppl.docx]
